# Rosai Dorfman Disease in Mandible: A Rare Case Report

**DOI:** 10.30476/dentjods.2022.95184.1844

**Published:** 2023-06-01

**Authors:** Hassan Mirmohammad Sadeghi, Roozbeh Azadi, Mehrdad Dehghanpour Barouj

**Affiliations:** 1 Dept. of Oral and Maxillofacial Surgery, Shahid Beheshti University of Medical Sciences, School of Dentistry, Tehran, Iran; 2 Student of Dentistry, Shahid Beheshti University of Medical Sciences, School of Dentistry, Tehran, Iran

**Keywords:** Rosai Dorfman disease, Sinus histiocytosis, Hematologic Disease, Pathology, Surgery

## Abstract

Rosai Dorfman disease is generally defined as a massive bilateral painless cervical lymphadenopathy accompanied with both fever and leukocytosis with neutrophilia. Additionally, it may possibly be associated with polyclonal hypergammaglobulinemia, reversal of CD4/CD8 ratio, the elevated erythrocyte sedimentation rate (ESR), microcytic anemia, and thrombocytosis. Rosai-Dorfman disease is known as a benign self-limiting disease, so no treatment is required in many cases, although it causes death in some cases by involving vital organs like kidney. The treatment is required when there is a life-threatening situation such as airway obstruction or involvement of vital organs such as kidney, liver, and lower respiratory tract. The required treatment choices include steroid therapy, chemotherapy, radiotherapy, and surgery. Surgical treatment is performed for bulk removal to resolve the obstruction caused by the mass as well as taking biopsy for the definite histopathologic diagnosis of disease. A 26-year-old man was referred to oral and maxillofacial surgery (OMFS) clinic of Taleghani hospital with chief complaints of pain and swelling of left submandibular space. According to the patient himself, the swelling had been started three months earlier. After rejecting dental source of the lesion, we decided to remove the mass by excisional biopsy concerning the patient’s discomfort. Histopathology report verified Rosai Dorfman disease as definite diagnosis of the mass.

## Introduction

Sinus histiocytosis with a massive lymphadenopathy (SHML) was firstly introduced and described by Rosai and Dorfman in 1969. Accordingly, it is also known as Rosai-Dorfman disease (RDD) [ 1 - 2 ]. This disease is generally defined by a massive bilateral painless cervical lymphadenopathy accompanied with both fever and leukocytosis with neutrophilia [ 1 , 3 - 4 ]. Additionally, it may possibly be associated with polyclonal hypergammaglobulinemia, reversal of CD4/CD8 ratio, the elevated ESR, microcytic anemia, and thrombocytosis [ 5 - 10 ]. RDD is histologically defined as lymphatic sinus dilatation caused by histiocyte proliferation that is very similar to a malignant neoplasm, despite its benign origin. Correspondingly, it usually shows slightly higher incidence rate in male patients [ 3 , 11 - 12 ]. It can occur in all age groups, but it more tends to affect children than adults [ 13 ]. Although it seems to be idiopathic, it is a disorder of the mononuclear phagocyte and an immunoregulatory effector (M-PIRE) system [ 14 ]. The altered immune responses and infectious agents such as Varicella Zoster, Herpetic viruses, Epstein-Barr, Cytomegalovirus, Brucella, and Klebsiella, are also announced as possible reasons for the incidence of RDD [ 10 ].

In about 43% of cases, RDD occurs in extra nodal form without any lymphadenopathy making this disease hard to be differentiated from other similar conditions [ 3 , 15 - 18 ]. Of note, the extra nodal form shows poorer prognosis compared to the nodal form [ 18 ]. Soft tissue of head and neck regions and the paranasal sinuses and the nasal cavity are the most common sites that are involved in this disease [ 16 , 19 ]. Some of the case reports of RDD lesions have been mentioned in (Table 1) briefly. Analysis of the same cases has shown the similar approach of surgical removal and steroid therapy for this lesion, although the location of RDD in our case is very rare and unique.

**Table 1:** Review of other articles related to Dorfman's disease in the jaw and face

	Location of the lesion	Treatment	Follow-up (months)
Ünal *et al*.[ 32 ] 2004	A lobulated, mobile, and firm submandibular mass (5x2 cm)- smooth surfaced masses on the left side of nasal septum as well as left nasal floor – a soft and smooth subglottic mass – several small masses among tracheal lumen	Surgical excision of all the masses	3
Goodnight *et al*. [ 8 ] 1996	A 5*5-cm multi-lobulated, mildly tender, enlarging parotid mass	Superficial and partial deep lobe parotidectomy	12
Montgomery *et al*. [ 33 ] 1992	A 1-cm mass in face	Local excision	-
Hazarika *et al*.[ 9 ] 2000	A solitary 0.5*0.5-cm Delphian node in left subglottic area	Surgical excision followed by steroids	2
Lai *et al*.[ 34 ] 2000	Bilateral nasal masses on cavity + anterior subglottic swelling + prominent nodes up to 2-cm in the left submandibular region and left posterior triangle apex	Excisional biopsy with CO_2_ laser and laryngeal microdebrider	<1
La Barge *et al* [ 25 ] 2008	Diffuse enlargement of submandibular glands	Surgical removal	-
	The bilaterally enlarged submandibular glands		
	Bilateral parotid masses		
Wenig *et al*. [ 16 ] 1993	Painless mass in the submandibular gland for 6 months	Surgical excision	4-96
	A painless mass in the parotid gland	Surgical excision	
Raslan *et al*. [ 26 ] 2011	Bilateral masses in neck lymph nodes (LN), submandibular glands and maxillary, ethmoidal, and nasal cavities	Steroids	1-75
	Bilateral lacrimal gland masses, right parotid, and neck LN enlargement	Chemotherapy, steroids	
	Bilateral LN enlargement and submandibular enlargement	steroids	

The characteristic of histopathologic finding for RDD is emperipolesis that is phagocytosis of lymphocytes, plasma cells, erythrocytes or polymorphonuclear (PMN) leukocytes by histiocytes, which is distinguished by large histiocyte-like cells, called Rosai-Dorfman (RD) cells [ 1 , 3 ]. The clinical course of RDD is unidentified, with episodes ranging from spontaneous regression to the protracted periods of stable lymphadenopathy as well as the less common observation of progressive lymphadenopathy occasionally with some subsequent recurrences [ 7 , 20 ]. The treatment plans proposed for RDD vary from steroid therapy, antibiotics, radiotherapy, chemotherapy, surgery to the combination of these methods; however, no definite treatment has been introduced for this disease yet [ 3 ]. Due to the chronic, relapsing nature of this disease, treatment is only necessary in persistent or worsening cases [ 21 ]. Of note, it can be mortal by infiltration into vital organs [ 22 ].

## Case Presentation

**Figure 1:**
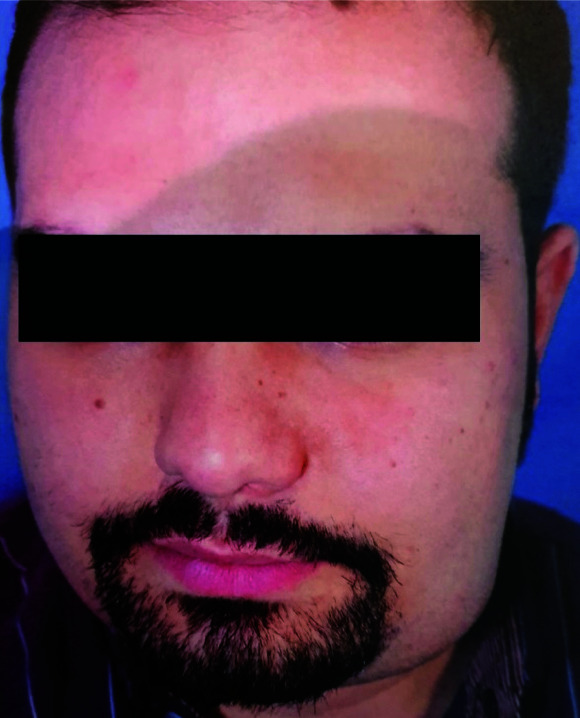
pre-operative photo of patient showing swelling of left submandibular space

A 26-year-old man was referred to Oral and Maxillofacial Surgery Clinic of Taleghani hospital with chief complaints of pain and swelling of left submandibular space. According to the patient himself, the swelling had been started 3 months earlier (Figure 1). 

**Figure 2:**
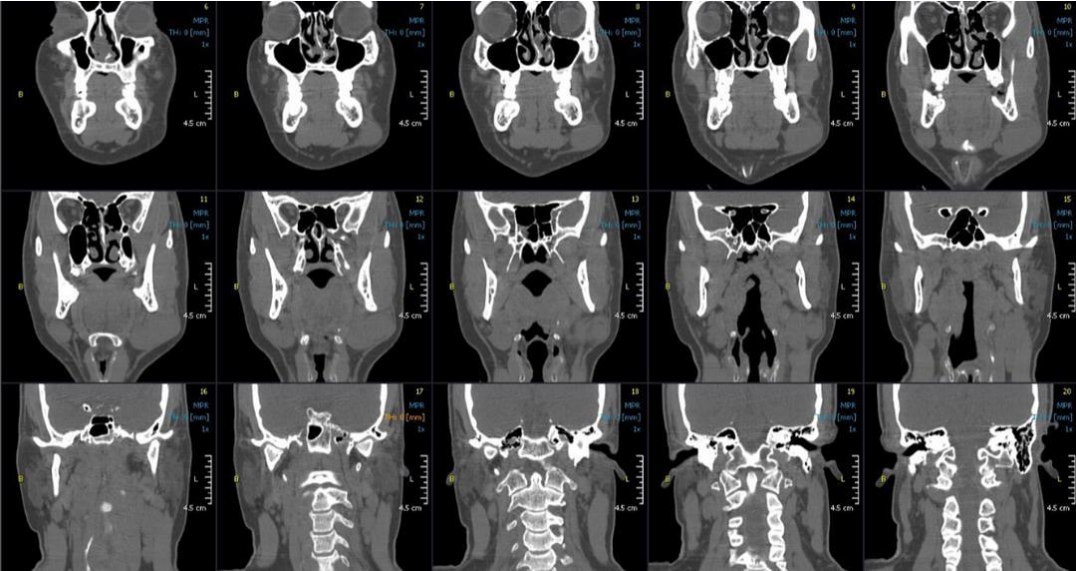
CT scan of patient's head and neck revealing two enhancing radiolucent masses in submandibular spaces of patient, especially in left side

**Figure 3:**
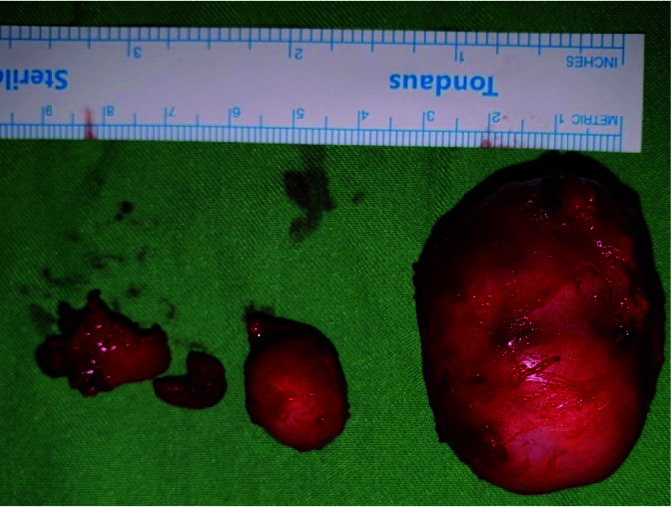
Excisional biopsy of the left submandibular lesions

**Figure 4:**
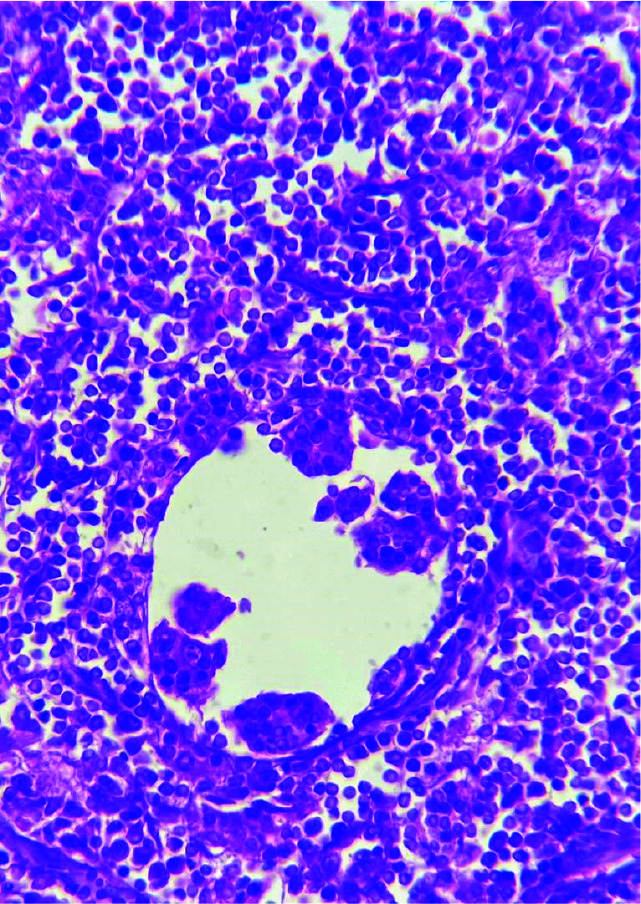
Microscopic view of Rosai-Dorfman cells, which are big histiocyte-like cells fored by phagocytosis of lymphocytes, plasmocytes, erythrocytes or polymorphonuclear (PMN) cells by histiocytes

He declared no former history of swelling or tooth pain in this area. After visiting a general dentist, Antibiotics were prescribed and used, and swelling disappeared after a week. After about 2 weeks, the swelling reoccurrence was observed at the same place. Thereafter, the extraction of left mandibular wisdom teeth was performed; however, the swelling did not decrease in size. The patient visited ENT faculty because of a mass observed in nasal passage and subsequently a fine needle aspiration (FNA) was performed for him. The pathology report showed a chronic inflammation associated with fibrous granulation reaction. The patient represented history of fever and weight loss in the past 2 months. Moreover, he had no history of smoking, but he stated alcohol usage occasionally. As well, he had a history of asthma when he was 15 years old. His family had no history of the same problem. Intra oral examination did not show any dental or mucosal defects. The wisdom tooth socket had shown normal healing process. Hematological tests such as complete blood check (CBC); blood sugar profile and biochemistry tests had shown normal values and no considerable abnormalities were detected. In computed tomography (CT) scan of head and neck regions (Figure 2), two enhancing masses were seen at left submandibular, lateral to the gland (45*30 and 16*10mm in diameter). Another mass was also observed at right side (26*27mm in diameter). Lymphadenopathy exists at level 2 and 3 at both left (19*27mm) and right sides (9mm). In abdominal CT scan, lymphadenopathy was visible in mesentery, and sonography report manifested an ovoid hypo-hetero eco lesions with well-defined borders observed in left and right jugular spaces with an internal vascularity seemed to be lymphadenopathy. Eventually, we decided to remove the pathologic lesion by excisional biopsy. Our differential diagnoses included a wide range of possible conditions that can cause such a mass in indicated area including (1) lymphoma and leukemia (2), Langerhans cell histiocytosis (LCH) (3), Tuberculosis or sarcoidosis or leprosy, and (4) Branchial cleft cysts (Figure 3). The operation was performed by passing one week from the patient’s first visit and we extracted a 4*4*2cm mass from his left submandibular space. Accordingly, the pathology report of resected mass described it as RDD that was an unexpected diagnosis. For definite diagnosis and rejecting other possibilities immunohistochemistry (IHC), staining for CD1 and S100 had been performed on the sample that confirmed our diagnosis. We decided not to remove the right lesion due to its smaller size, but a minimum dose of Prednisolone (5 mg that gradually reduced to 2.5 mg) was prescribed and routine follow up scheduled for the patient. The patient’s condition was followed up for 2 years and no recurrence of lesion had been observed (Figure 4). 

## Discussion

In 1969, Rosai and Dorfman have introduced a newly recognized benign entity and later, they introduced more cases with this disorder and called it RDD [ 1 - 2 ]. The more accurate name of this disorder is sinus histiocytosis with a massive lymphadenopathy (SHML). Although the etiology of RDD is not certainly defined yet, infectious agents, autoimmune disorders, suppression of immune system, and malignancies have been reported as the causes of the disease [ 1 - 2 , 16 ]. In the present study, our patient declared a history of asthma that can be related to RDD. 

SHML can definitely be diagnosed histopathologically. The histopathologic diagnostic feature of the disease is emperipolesis, which is phagocytosis of lymphocytes, plasmacytes, erythrocytes, or PMN leukocytes by histiocytes (also called lymphophagocytosis). In addition to emperipolesis, Russell bodies, and foamy histiocytes are mostly seen in the intra-nodal form of the disease [ 1 - 2 , 16 , 23 ]. Immunohistochemical features of RDD are positive S-100 alpha-antichymotrypsin as well as CD1a and CD68 antigens [ 10 , 23 - 24 ]. The most common sign of the disease is painless cervical lymphadenopathy [ 25 - 26 ], which is represented as a cervical mass. However, in our case, pain was represented in the swelling zone. This may lead to various differential diagnoses such as infectious, lymphomatous, and nonlymphomatous causes, while massive painless bilateral lymph node enlargement of neck, especially in younger patients and adolescents, greatly increased the chance of detecting RDD in the differential diagnosis [ 27 - 29 ]. As well, the associated lymphadenopathy of RDD is detectable in magnetic resonance imaging (MRI), medical CT, sonography, and scintigraphy. The sonographic pattern of the involved nodes is similar to malignancies [ 28 ]. The increased uptake of gallium and raised metabolism of F-FDG on PET scan are detectable in nuclear imaging [ 11 , 29 - 30 ]. Although the mass is visible using paraclinical imaging techniques, histopathological analysis is necessary for the definite diagnosis of RDD [ 25 , 28 ]. RDD is known as a benign self-limiting disease, so no treatment is needed in many cases [ 31 , 33 - 34 ], although some cases of death by vital organs like kidney [ 22 ], have been reported. The treatment is required when there is a life-threatening situation such as airway obstruction or the involvement of vital organs such as kidney, liver, and lower respiratory tract [ 3 ]. The required treatment choices include steroid therapy, chemotherapy, radiotherapy, and surgery [ 9 ]. Surgical treatment is performed for bulk removal to resolve the obstruction caused by the mass as well as taking biopsy for the definite histopathologic diagnosis of disease. FNA and lymph node removal can also be helpful in better diagnosing RDD [ 9 ], as we used in our case before the surgical excision.

An informed consent of the patient was obtained to use the information and photos of the patient in the article.

## Conclusion

RDD is a rare, benign, and self-limiting disease. Although few cases of RDD have been reported up to now, the similarity of signs and symptoms of RDD, tuberculosis, and malignant tumors like lymphomas, makes the correct diagnosis of the disease more challenging. Histopathology is the definitive way for diagnosing RDD by detecting emperipolesis. Treatment is not necessary in benign cases, although in life-threatening cases, surgical treatment is needed to avoid mortality. The recurrence of disease is probable; therefore, routine follow-up is recommended.

## Conflict of Interest

Authors declare that there are no conflicts of interest in this study. 
